# NPY1R exerts inhibitory action on estradiol-stimulated growth and predicts endocrine sensitivity and better survival in ER-positive breast cancer

**DOI:** 10.1038/s41598-022-05949-7

**Published:** 2022-02-04

**Authors:** Raksha Bhat, Hariprasad Thangavel, Noor Mazin Abdulkareem, Suhas Vasaikar, Carmine De Angelis, Leon Bae, Maria Letizia Cataldo, Sarmistha Nanda, Xiaoyong Fu, Bing Zhang, Rachel Schiff, Meghana V. Trivedi

**Affiliations:** 1 Department of Pharmacy Practice and Translational Research, University of Houston College of Pharmacy, 4849 Calhoun Rd, Houston, TX 77204 USA; 2 Department of Pharmacological and Pharmaceutical Sciences, University of Houston College of Pharmacy, Houston, TX 77204 USA; 3 Lester and Sue Smith Breast Center, Baylor College of Medicine, Houston, TX 77030 USA; 4 Department of Clinical Medicine and Surgery, University of Naples Federico II, 80131 Naples, Italy; 5 Dan L. Duncan Comprehensive Cancer Center, Baylor College of Medicine, Houston, TX 77030 USA; 6 Department of Molecular and Cellular Biology, Baylor College of Medicine, Houston, TX 77030 USA; 7 Department of Molecular and Human Genetics, Baylor College of Medicine, Houston, TX 77030 USA; 8 Department of Medicine, Baylor College of Medicine, Houston, TX 77030 USA

**Keywords:** Cancer, Biomarkers

## Abstract

G Protein-Coupled Receptors (GPCRs) represent the largest superfamily of cell-surface proteins. However, the expression and function of majority of GPCRs remain unexplored in breast cancer (BC). We interrogated the expression and phosphorylation status of 398 non-sensory GPCRs using the landmark BC proteogenomics and phosphoproteomic dataset from The Cancer Genome Atlas. Neuropeptide Y Receptor Y1 (NPY1R) gene and protein expression were significantly higher in Luminal A tumors versus other BC subtypes. The trend of NPY1R gene, protein, and phosphosite (NPY1R-S368s) expression was decreasing in the order of Luminal A, Luminal B, Basal, and human epidermal growth factor receptor 2 (HER2) subtypes. *NPY1R* gene expression increased in response to estrogen and reduced with endocrine therapy in estrogen receptor-positive (ER+) BC cells and xenograft models. Conversely, NPY1R expression decreased in ER+ BC cells resistant to endocrine therapies (estrogen deprivation, tamoxifen, and fulvestrant) in vitro and in vivo. NPY treatment reduced estradiol-stimulated cell growth, which was reversed by NPY1R antagonist (BIBP-3226) in ER+ BC cells. Higher NPY1R gene expression predicted better relapse-free survival and overall survival in ER+ BC. Our study demonstrates that NPY1R mediates the inhibitory action of NPY on estradiol-stimulated growth of ER+ BC cells, and its expression serves as a biomarker to predict endocrine sensitivity and survival in ER+ BC patients.

## Introduction

Approximately 75–80% of breast cancer (BC) are estrogen receptor-positive (ER+)^1^, indicating their growth stimulation by estrogen. Endocrine therapies, which include tamoxifen (Tam), aromatase inhibitors, luteinizing hormone-releasing hormone receptor agonists, and fulvestrant are the standard of care treatment options for ER+ BC^2–5^. The use of endocrine therapies significantly improves long-term outcomes in early- and advanced-stage ER+ BC. However, intrinsic or acquired resistance occur frequently with all endocrine therapies, resulting in disease relapse and poor survival^6–9^. Therefore, the development of biomarkers to predict endocrine resistance and effective drug targets to overcome endocrine resistance is of utmost importance in ER+ BC.

G protein-coupled receptors (GPCRs) are highly ‘druggable’ targets. Nearly 30–50% of all Food and Drug Administration (FDA)-approved drugs target various GPCRs or their pathways and are often used to treat various chronic diseases due to their excellent safety profile^10–15^. Several GPCRs and/or their ligands have been implicated in cancer cell biology playing a role in multiple cellular processes including cell growth, proliferation, survival, invasion, and migration^16–23^. But, GPCRs have not been systematically explored as biomarkers or drug targets in BC. While genetic aberrations in kinases and transcription factors are thought to contribute to cancer development^24^, these alterations are not very common in GPCRs. However, posttranslational modification such as phosphorylation and ubiquitination, which affects GPCR activity^25–28^, might be highly relevant for controlling various cellular processes important in cancer biology.

The goal of this study was to interrogate the altered expression and phosphorylation of various GPCRs in BC using provisional proteomic and phosphoproteomic data from National Cancer Institute’s Clinical Proteomic Tumor Analysis Consortium (NCI-CPTAC). Previously, phosphoproteome profiling of breast cohort by Mertins et al. have identified a subgroup with differential GPCR activity and role in BC^29^. Here, we evaluated the proteome and phosphoproteome data of 398 non-sensory GPCRs [International Union of Basic and Clinical Pharmacology/British Pharmacological Society (IUPHAR/BPS) Guide to Pharmacology, IUPHAR v1.0] using the landmark The Cancer Genome Atlas (TCGA) BC proteogenomic dataset. We also assessed the expression of Neuropeptide Y Receptor Y1 (NPY1R), the topmost hypo-phosphorylated (at S368s) GPCR in BC patient samples. We found that NPY1R serves as a predictor of endocrine sensitivity and of long-term outcomes in ER+ BC.

## Methods

### Selection of proteomic and phosphoproteomic data

The published datasets from the TCGA BC proteogenomics analysis by Mertins et al. were used in this study^29^. The datasets included 77 primary breast tumors for which comprehensive, quantitative mass-spectrometry-based proteomic and phosphoproteomic analyses were performed. The data were represented as protein-level or phosphosite-level log2-transformed iTRAQ (Isobaric tags for relative and absolute quantitation) ratio. The iTRAQ ratio was calculated using the median of all PSM (peptide spectrum match) level ratios contributing to a protein subgroup or phosphosite^29^. The data were normalized where log-ratios centered around zero, and up- or down-regulated proteins or phosphosites were characterized in comparison to the reference (pooled samples). GPCRs from the proteomics data (18 identified proteins), and phosphoproteomics data (136 identified phosphosites) were selected for further analysis (The IUPHAR/BPS Guide to Pharmacology, IUPHAR v1.0; https://www.guidetopharmacology.org/).

### RNA Expression data for BC patients and cell lines

The Firehose Genome Data Analysis Center (GDAC) portal was used to download processed TCGA RNA-Seq data (Illumina HiSeq)^30^. The Log2 transformed normalized RSEM count data for tumor samples (*n* = 1093) were extracted. The PAM50 annotation was used for samples with Luminal A (LumA) (*n* = 415), Luminal B (LumB) (*n* = 176), Basal (*n* = 136) and human epidermal growth factor receptor 2 (HER2) (*n* = 65) as previously described^31^. Further, the paired samples (tumor and normal) were extracted (*n* = 112) from the Illumina HiSeq dataset. NPY1R expression across TCGA cancer cohort was obtained from human protein atlas (HPA)^32^. BC subtype-specific expression of NPY1R was analyzed using the METABRIC dataset^33^. The Broad Institute Cancer Cell Line Encyclopedia (CCLE) database was used to analyze mRNA expression of NPY1R across BC cell lines of different molecular subtypes^34^.

### NPY1R expression from published affymetrix microarray and RNA-seq data

NPY1R gene expression was assessed in the following publicly available datasets from our group: (i) time-dependent expression with estradiol-treatment in MCF7, T47D, and BT474 cells^35–37^; (ii) in response to various endocrine therapies in MCF7 and T47D cells; (iii) parental cells and their estrogen deprivation-resistant (EDR), Tam-resistant (TamR), and fulvestrant-resistant (FulR) derivatives of MCF7, T47D, and ZR75-1 BC cells grown in vitro^38^ and (iv) in xenograft tumors generated from MCF7 parental, EDR, TamR, and FulR derivatives in viv ^36,37^.

### Drugs

17β-estradiol (E2) (catalog #E2758) and Neuropeptide Y (NPY) human (catalog #N5017) were purchased from Sigma-Aldrich, USA. BIBP-3226 trifluoroacetate (catalog #2707) was purchased from Tocris Bioscience.

### Cell lines and establishment of resistant lines

The human BC cells MCF7 (MCF7L, originally from Dr. Marc Lippman’s lab) and T47D (purchased from ATCC, Manassas, VA, USA) were maintained in RPMI-1640 medium (Lonza, Walkersville, MD) supplemented with 10% fetal bovine serum (FBS) and 1% Penicillin–Streptomycin-Glutamine (PSG). The endocrine resistant derivatives, EDR of MCF7 and T47D cells were generated using a previously published method^39^. These cells were grown in RPMI-1640 medium (Lonza, Walkersville, MD) supplemented with 10% Charcoal stripped serum and 1% PSG.

### Quantitative polymerase chain reaction (qPCR)

NPY1R gene expression was assessed in MCF7 and T47D cells grown in charcoal-stripped media were treated with E2, estrogen deprivation (ED), or ED + tamoxifen (Tam). qPCR analysis was performed as previously described^40^. NPY1R primers-probe (cat# 4448892, Assay ID: Hs00702150_s1, ThermoFisher) were used. The fold change data were plotted as relative quantification RQ (2^(− ddct)^)^41^.

### Immunoblotting

NPY1R protein expression was evaluated in parental and EDR derivatives of MCF7, T47D, and ZR75-1 cells by immunoblotting. Briefly, cells were lysed using cell lysis buffer (catalog# 9803S, Cell Signaling Technology) and cell membrane proteins were extracted using Mem-PER™ Plus Membrane Protein Extraction Kit (catalog #89842, ThermoFisher Scientific) as per the manufacturer’s instructions. NPY1R expression was detected using anti-NPY1R primary antibody (catalog #NBP1-59008, Novus Biologicals). Ponceau staining was used to assess equal loading of cell membrane proteins.

### Cell proliferation assay

The cell growth and viability were assessed using the 3-(4,5-dimethylthiazol-2-yl)-2,5-diphenyltetrazolium bromide (MTT) assay kit (catalog #30-1010K™, ATCC®). Briefly, MCF7 and T47D were grown with or without antagonist BIBP-3226 (1 µM) and plated in triplicates (10,000–15,000 cells/well) in 96-well tissue culture plates. After overnight attachment, the cells were treated with vehicle or E2 immediately followed by the addition of NPY (100 nM) in 15 min. The cell proliferation was determined after 72 h by following the MTT kit protocol.

### Survival analysis

Kaplan–Meier analysis was performed using an online tool for meta-analysis of public microarray datasets^42^ (https://www.kmplot.com/analysis/). The impact of NPY1R expression on relapse-free survival (RFS) and overall survival (OS) in patients with ER+ and ER− tumors (defined by IHC) were analyzed by assessing the effects of 22,277 genes on survival in 2422 BC patients. METABRIC dataset was used to predict BC specific survival (BCSS) of LumA and LumB BC patients with high and low NPY1R expression.

### Statistical analysis

The statistical analyses presented in the manuscript were performed using R version 3.4.0^43^ (https://www.r-project.org/) and GraphPad Prism 9.3.1 (https://www.graphpad.com/). The t-test (paired) was used to compare paired samples otherwise stated. ANOVA analysis was performed on PAM50 subtype data and further post-hoc analysis was performed using Tukey’s test. All analyses were presented with statistical *p*-value significance by appropriate methods. The probability of survival was estimated by the Kaplan–Meier method.

## Results

### NPY1R is highly expressed and hyper-phosphorylated in LumA BC patients

**Figure 1:**
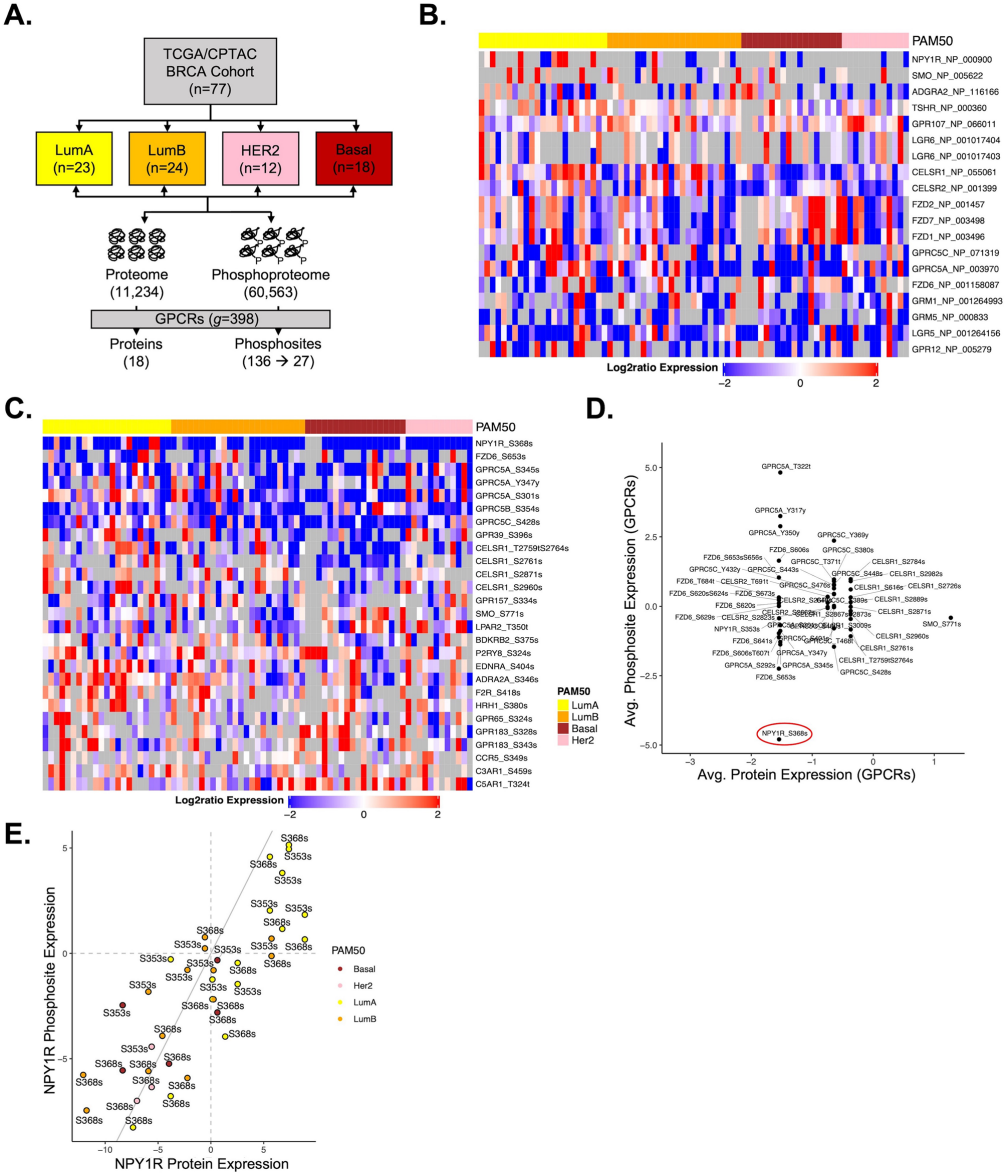
GPCR proteomic and phosphoproteomic analysis identified NPY1R as a potential GPCR target in luminal BC. (**A**) NCI-CPTAC breast cohort of 77 patients consisting of 23 LumA, 24 LumB, 12 HER2 and 18 basal-like tumors. The mass spectrometry based iTRAQ method identified a total 11,234 proteins and 60,563 phosphosites in 77 samples of which 18 proteins and 136 sites (27 sites with less than 50% missing value) overlapped with GPCRs. (**B**) The 18 proteins that were quantified: NPY1R, SMO, ADGRA2, TSHR, GPR107, LGR5, LGR6, CELSR1, CELSR2, FZD1, FZD2, FZD6, FZD7, GPRC5A, GPRC5C, GRM1, GRM5, and GPR12. (**C**) The 27 phosphosites corresponding to 21 proteins were NPY1R, FZD6, GPRC5A, GPRC5B, GPRC5C, GPR39, GPR65, GPR157, GPR183, CELSR1, SMO, LPAR2, BDKRB2, P2RY8, EDNRA, ADRA2A, F2R, HRH1, CCR5, C3AR1, and C5AR1. (**D**) Average NPY1R protein and phosphosite expression showed hypophosphorylation at S368s in overall samples but (**E**) hyperphosphorylation in lumA subtype. The expression values ranging from -2 to 2 (blue to red) indicate aggregated iTRAQ log-ratios for each sample, which are normalized by subtracting the mean of all values. Each row represents an individual GPCR and each column is a patient sample.

To study GPCRs’ expression in BC, we used NCI-CPTAC’s proteomics and phosphoproteomics data^29^ which was previously characterized by TCGA with publicly available corresponding genomic data^36^. NCI-CPTAC breast cohort consisted of QC metrics qualified 77 patients, 23 LumA, 24 LumB, 12 HER2 (ERBB2)-enriched and 18 Basal-like tumors (Fig. 1A). In 77 samples, an isobaric peptide labeling approach (iTRAQ) identified a total 11,234 proteins and 60,563 phosphosites. Among them, we observed 18 GPCRs and 136 phosphosites overlapped with 398 GPCRs obtained from IUPHAR (The IUPHAR/BPS Guide to Pharmacology, IUPHAR v1.0). We selected 18 proteins and 27 sites with less than 50% missing value for subsequent analyses in this study. The following 18 GPCRs were quantified: NPY1R, SMO, ADGRA2, TSHR, GPR107, LGR5, LGR6, CELSR1, CELSR2, FZD1, FZD2, FZD6, FZD7, GPRC5A, GPRC5C, GRM1, GRM5, and GPR12 (Fig. 1B). The 21 GPCRs corresponding to 27 phosphosites were: NPY1R, FZD6, GPRC5A, GPRC5B, GPRC5C, GPR39, GPR65, GPR157, GPR183, CELSR1, SMO, LPAR2, BDKRB2, P2RY8, EDNRA, ADRA2A, F2R, HRH1, CCR5, C3AR1, and C5AR1 (Fig. 1C). Among 21 phosphoproteins, NPY1R (90%; n = 64/71) was largely hypo-phosphorylated followed by GPRC5A (84%; n = 60/71), GPRC5B (77%; n = 60/77), and GPRC5C (67%; n = 48/71) comparing across the sample cohort^29^. Average NPY1R protein and phosphosite expression showed greater hypophosphorylation at S368s in overall BC samples (Fig. 1D) but was hyperphosphorylated in LumA subtype samples (Fig. 1E). NPY1R expression was higher in BC compared to other 16 cancers across the TCGA cancer cohort obtained from HPA (Supplementary Fig. 1).

### NPY1R gene and protein expression and phosphorylation status is subtype-specific in BC Patients

**Figure 2:**
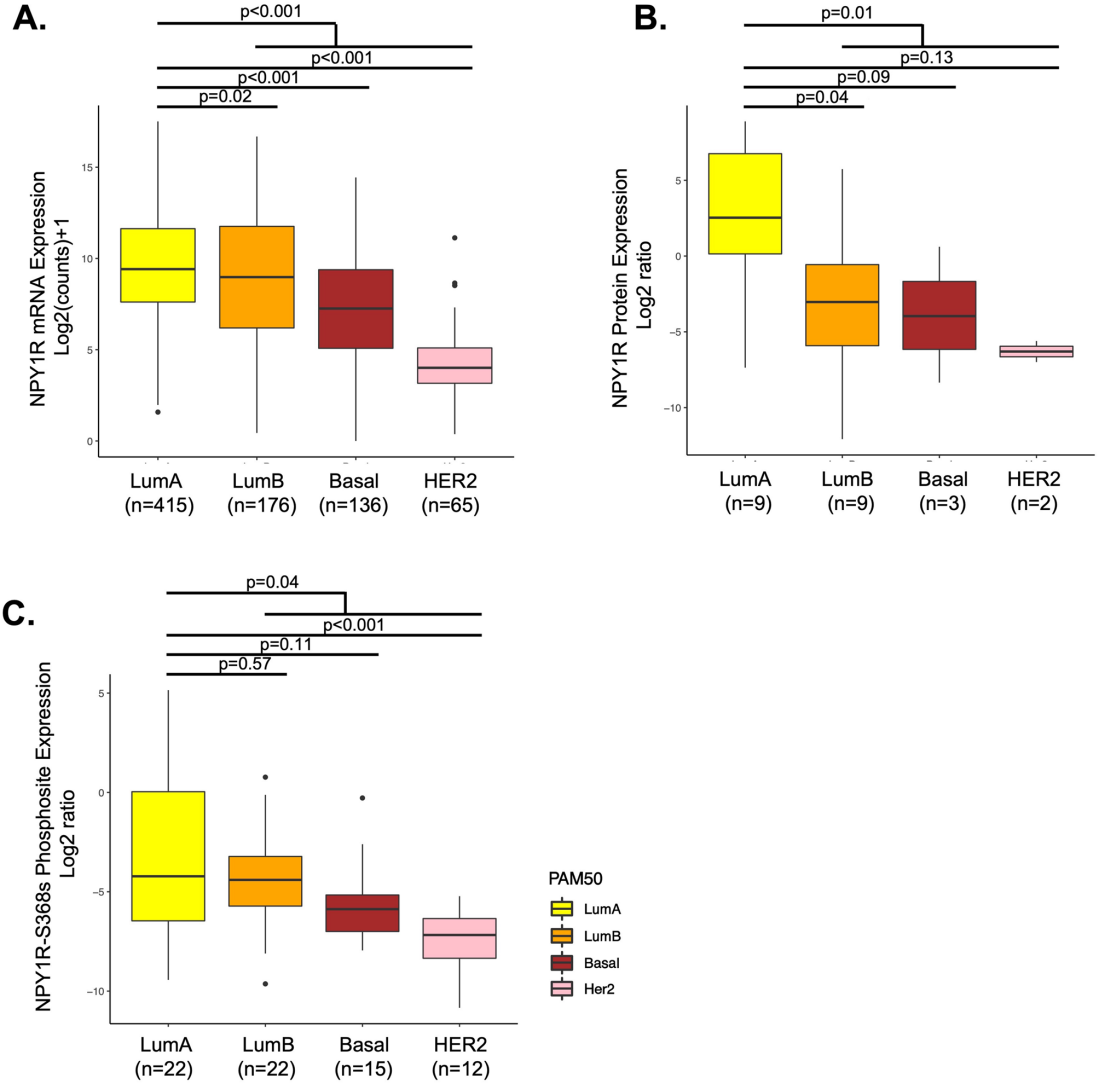
NPY1R expression at mRNA, protein and phosphosite level across BC subtype. (**A**) NPY1R RNA expression in TCGA breast cohort (n = 792), (**B**) protein expression in NCI-CPTAC samples (n = 23), and (**C**) NPY1R-S368s phosphosite expression in corresponding NCI-CPTAC dataset (n = 71). PAM50 subtypes were shown as LumA (yellow), LumB (orange), HER2 (pink) and Basal (brown). Statistical analyses were performed using one-way ANOVA with post-hoc Tukey Test. *indicates p < 0.05.

To explore the subtype-specific enrichment in BC, we compared the RNA, protein, and phosphosite expression of NPY1R across 4 BC subtypes (LumA, LumB, HER2, Basal). In TCGA-BRCA dataset (n = 817) with known PAM50 information^31^, we observed differential relative abundance of NPY1R gene transcripts in each of the BC subtypes. The trend was found to be decreasing in the order of LumA, LumB, Basal, and HER2 subtype. Both LumA and B showed a relatively high abundance of *NPY1R* gene transcripts (p < 0.001, Tukey test; Fig. 2A). In METABRIC dataset (n = 1758), *NPY1R* gene expression was significantly higher in LumA (n = 679) and LumB (N = 461) BC patients (Supplementary Fig. 2). Similarly, in the CCLE dataset of BC cell lines, *NPY1R* gene expression was higher in LumA compared to other subtypes (Supplementary Fig. 3). Protein expression data from matched patients revealed significantly higher levels of NPY1R (p = 0.01) only in LumA compared to all other subtypes combined, implying a specific up-regulation mechanism for the overexpression of NPY1R that is limited to LumA subtype tumors (Fig. 2B). NPY1R phosphorylation at S368 was higher in LumA subtype compared to combination of other subtypes (p = 0.04) (Fig. 2C) and the trend was found similar to that seen on RNA level. There was no significant difference in NPY1R protein and phospho-protein between LumA and other individual BC subtypes, likely due to smaller sample size.

### NPY1R is an estrogen-responsive gene

**Figure 3:**
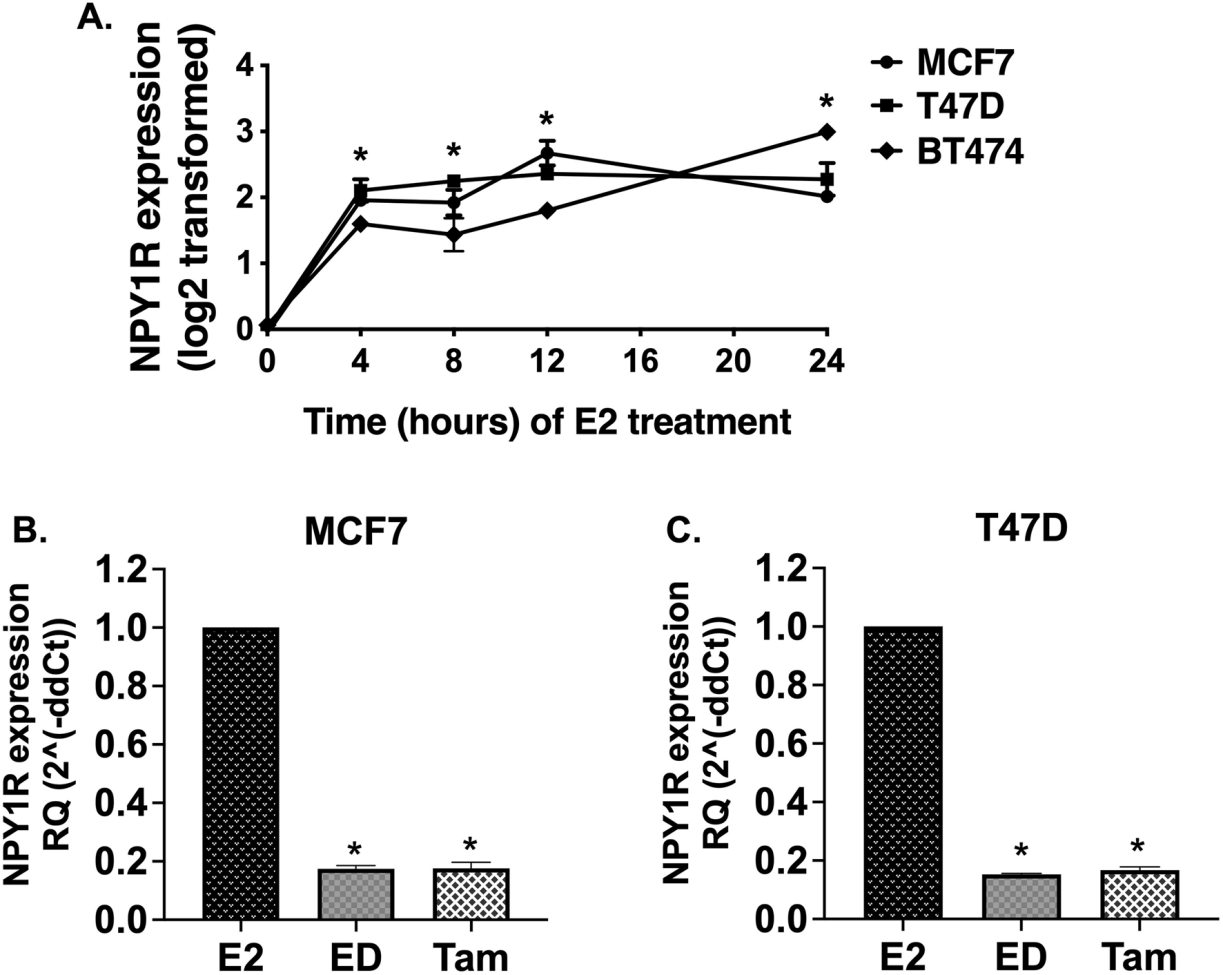
17β-Estradiol (E2) treatment induces NPY1R gene expression in a time-dependent manner and endocrine therapy downregulates NPY1R gene expression in ER+ BC cells. (**A**) Data presented is derived from microarray-based gene expression profiles from three ERα-positive breast cancer cell lines (MCF7, T47D and BT474) stimulated by (E2) in vitro over a time course 0–24 h. RNA was isolated, and the gene expression profiles were determined using Affymetrix Genechip Arrays. Arrays were normalized and compared using DNA-Chip Analyzer software (dChip), (http://www.dchip.org/) (GEO: GSE3834)^35^. Gene expression values were log-transformed. NPY1R probe: 205440_s_at. Each data point represents the mean of duplicates (BT474 at 12 h is an individual value). Error bars show the SD (automatically calculated by GraphPad prism). (**B**) MCF7 and (**C**) T47D cells grown in charcoal-stripped media were treated with E2, estrogen deprivation (ED), or ED + tamoxifen (Tam). Total RNA was extracted for qPCR analysis and the fold change data were plotted as RQ (2^(− ddct)^). *indicates statistically significant difference compared to 0 h time point or E2; *indicates p < 0.05 by One-way ANOVA, Dunnett’s test (n = 3).

Next, we evaluated NPY1R expression using our available datasets from BC cell line models in response to estrogen and endocrine therapy^35–37^. NPY1R gene expression was significantly upregulated in all 3 BC cells (MCF7, T47D, and BT474) in response to E2 treatment in a time-dependent manner (0–24 h) (p < 0.05, One-way ANOVA, Dunnett test) (Fig. 3A). Conversely, NPY1R gene expression was reduced after estrogen deprivation (ED) or with ED plus Tam in MCF7 and T47D cell models in RNA-seq analysis (Supplementary Fig. 4) as well as by qPCR (p < 0.05, One-way ANOVA, Dunnett test) (Fig. 3B,C).

### NPY1R expression is downregulated in endocrine-resistant BC

**Figure 4:**
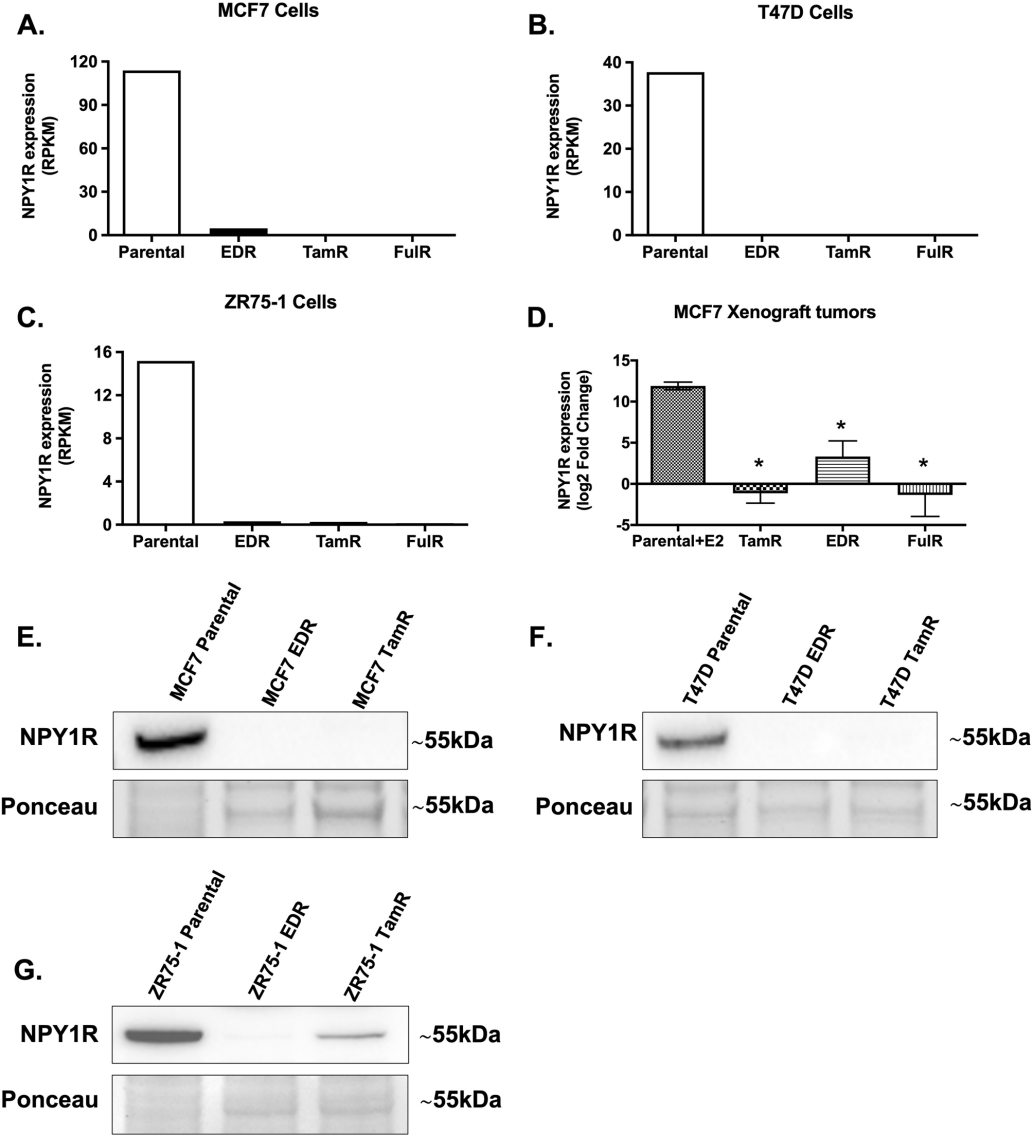
NPY1R gene and protein expression is impaired in endocrine-resistant derivatives of ER+ BC cells in vitro and downregulated in tamoxifen-resistant xenograft tumors in vivo. RNA-Seq data of parental and their estrogen deprivation-resistant (EDR), tamoxifen-resistant (TamR), and fulvestrant-resistant (FulR) derivatives of (**A**) MCF7, (**B)** T47D, and (**C**) ZR75-1 cells were interrogated^38^. Data were plotted as Reads Per Kilobase of transcript per Million mapped reads (RPKM). (**D**) U133plus2 Affymetrix Chip array data from MCF-7 tumors derivatives of EDR, TamR, and FulR, alongside parental tumors in presence of continued estrogen supplementation (E2) was evaluated and plotted as RNA Express-calculated signal intensity (log2 transformed fold change)^36,37^. *indicates p < 0.05 by One-way ANOVA, Dunnett’s test (n = 3–4). The protein expression of NPY1R was compared in MCF7, T47D and ZR75-1 parental cells to their EDR and TamR derivatives. Representative immunoblotting images showing the protein expression of NPY1R in (**E**) MCF7, (**F**) T47D and (**G**) ZR75-1 parental cells and their EDR and TamR derivatives. Ponceau staining was used to assess equal protein loading for visual assessment. The densitometric quantitation of the relative intensity of NPY1R to parental cells (from three independent experiments) is shown in Supplementary Fig. 5. Full-length blots are presented in Supplementary Fig. 5.

We also evaluated NPY1R expression in our available datasets from in vitro and in vivo BC models of endocrine resistance^36–38^. *NPY1R* gene expression was significantly reduced in EDR, TamR, and FulR derivatives of MCF7 (Fig. 4A), T47D (Fig. 4B), and ZR75-1 (Fig. 4C) ER+ BC cell line models compared to the parental cells (p < 0.05, One-way ANOVA, Tukey test). Similarly, *NPY1R* gene expression was downregulated in TamR, EDR, and FulR MCF7 xenograft tumors compared to parental MCF7 xenografts grown with continued E2 supplementation (p < 0.05, One-way ANOVA, Dunnett’s test) (Fig. 4D). Next, we compared the NPY1R protein expression on plasma membrane in MCF7, T47D, and ZR75-1 parental cells to their EDR and TamR derivatives. We found that NPY1R protein expression was diminished in EDR and TamR derivatives compared to their parental cells of MCF7 (Fig. 4E), T47D (Fig. 4F) and ZR75-1 (Fig. 4G) cell line models. The band at ~ 55 kDa was considered to be NPY1R, which was the prominent band in MCF7, T47D, and ZR75-1 parental cells, as reported before^44,45^. The full-length blots and the densitometric quantitation of the relative intensity of NPY1R to parental cells (from three independent experiments) are shown in Supplementary Fig. 5.

### Inhibition of E2-stimulated cell growth by NPY is mediated via NPY1R

**Figure 5:**
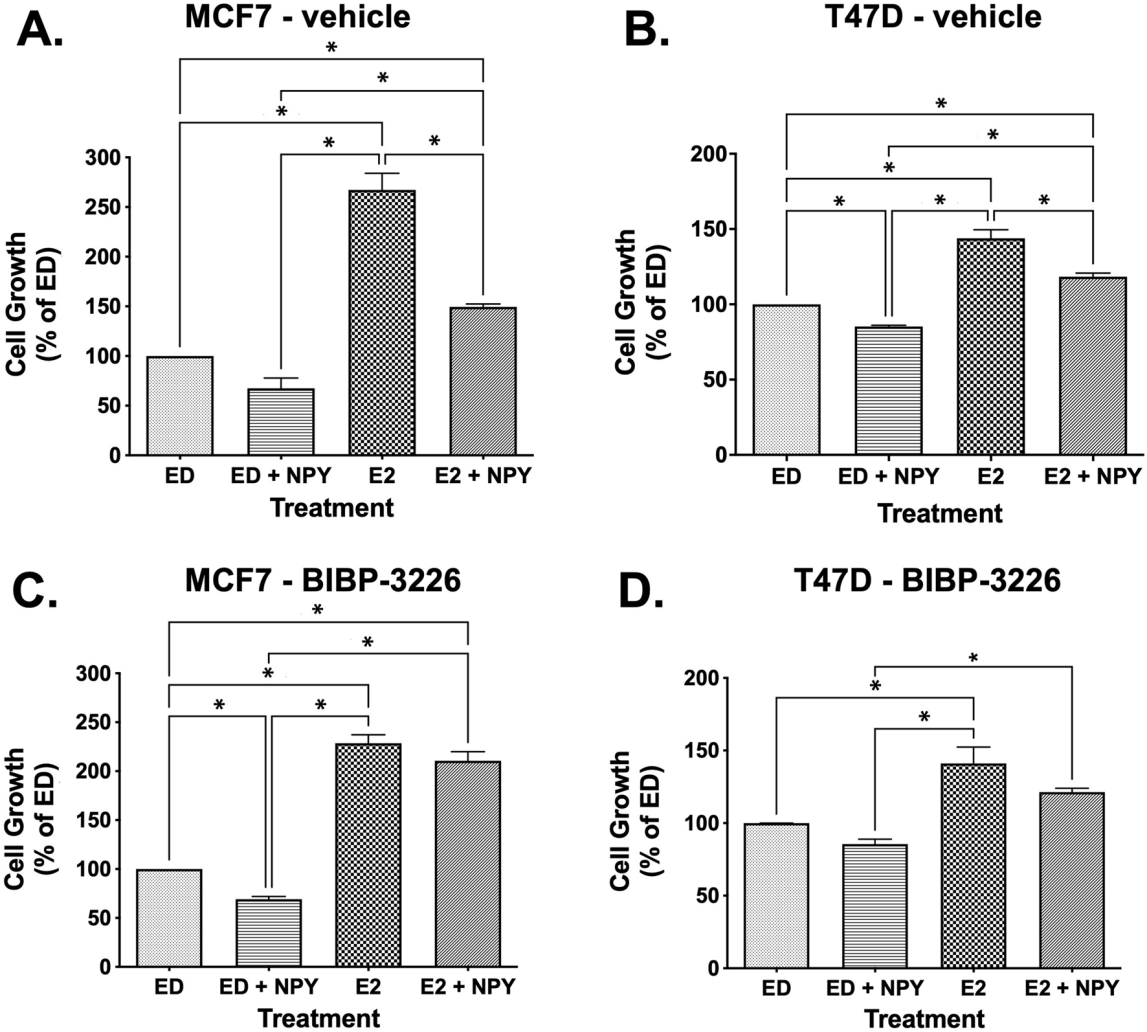
NPY-mediated inhibition of estradiol-stimulated cell growth is reversed by NPY1R antagonist BIBP-3226 in ER+ BC cells. MCF7 and T47D cells were treated with either vehicle (0.1% DMSO) (**A**, **B**) or 1 µM BIBP-3226 (**C**, **D**) and then plated in 96-well plates. After overnight attachments, the cells were treated with either vehicle (0.1% ethanol), 100 nM neuropeptide Y, 1 nM estradiol, or 100 nM NPY + 1 nM estradiol. The cell proliferation was determined using MTT assay after 72 h. *indicates p < 0.05, One-way ANOVA, Tukey test.

NPY significantly inhibited E2-stimulated cell growth in MCF7 and T47D cells (p < 0.05, One-way ANOVA, Tukey test) (Fig. 5A and 5B). The inhibitory effect of NPY on E2-stimulated cell growth was reversed by treatment with BIBP-3226, a selective non-peptide antagonist of NPY1R^46–50^, in MCF7 and T47D cells (Fig. 5C and 5D). Notably, BIBP-3226 had no significant effects on non-E2-stimulated cell growth in MCF7 and T47D cells (Supplementary Fig. 6).

### NPY1R is a prognostic marker in ER+ and LumA BC

**Figure 6:**
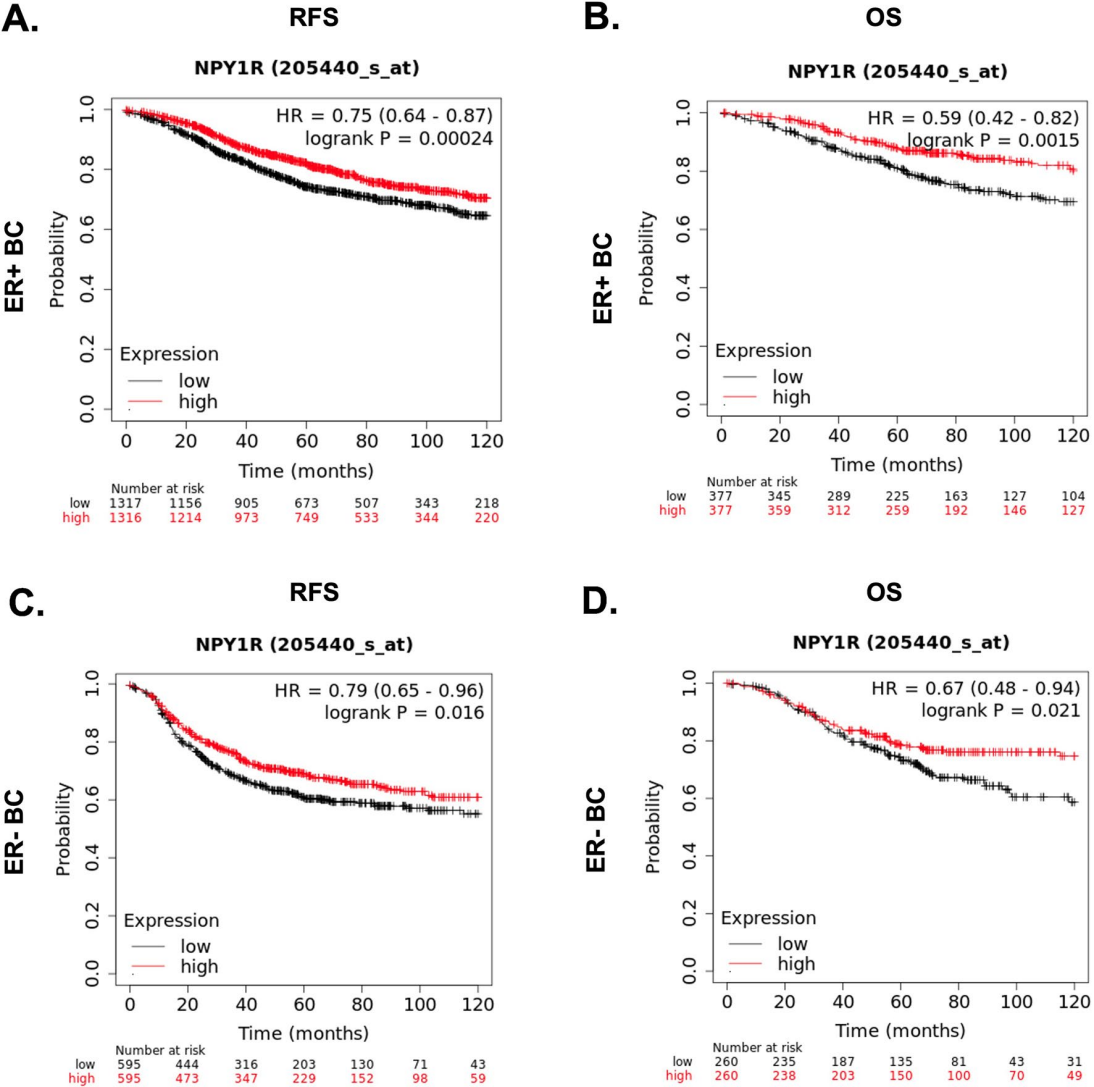
NPY1R expression predicts better relapse-free survival (RFS) and overall survival (OS) in ER+ BC patients. The impact of NPY1R expression on RFS (**A**, **C**) and OS (**B**,** D**) in ER+ (**A**, **B**) and ER− (**C**, **D**) BC patients was plotted using KMplotter, which assessed the effects of 22,277 genes on survival in 2422 BC patients. Red line: higher than median NPY1R expression; black line: lower than median NPY1R expression. *HR* hazard ratio. Log-rank P-value of < 0.05 was considered statistically significant.

Higher *NPY1R* gene expression in ER+ BC patients regardless of HER2 status predicted longer RFS [HR = 0.8 (range: 0.68–0.94, logrank P = 0.0072)] (Fig. 6A) and OS [HR = 0.62 (range: 0.43–0.89, logrank P = 0.009)] (Fig. 6B). In ER− BC patients, higher *NPY1R* gene expression was not predictive of RFS [(HR = 0.8 (range: 0.64–1.01, logrank P = 0.055) (Fig. 6C) but was associated with better OS [(HR = 0.61 (range: 0.38–0.97, logrank P = 0.034)] (Fig. 6D). In METABRIC dataset, high *NPY1R* expression predicted significantly longer BCSS in LumA (n = 678, logrank P = 0.0147) tumors but not in Lum B tumors (Supplementary Fig. 7).

## Discussion

In this study, we have evaluated the proteome and phosphoproteome data of 398 non-sensory GPCRs using the landmark TCGA BC proteogenomics dataset. NPY1R was the topmost hypo-phosphorylated (at S368s) GPCR in more than 90% of the samples tested. Among tumor samples, the LumA subtype had significantly higher NPY1R expression than all other BC subtypes. Interestingly, the trend of NPY1R gene and protein expression as well as phosphorylation at S368s were decreasing in the order of LumA, LumB, Basal, and HER2 subtypes. The up- and down-regulation of NPY1R gene expression in ER+ BC cells upon treatment with E2 and Tam or ED, respectively, confirmed NPY1R as an estrogen-responsive gene. In endocrine-resistant (TamR, EDR, and FulR) derivatives of ER+ BC cell line models, NPY1R expression remained significantly lower compared to the parental cells both in vitro and in vivo. In the publicly available dataset, higher NPY1R expression predicted better OS and RFS in ER+ and LumA BC patients. NPY treatment significantly reduced E2-stimulated cell growth in ER+ BC cells, which was reversed by NPY1R antagonist, BIBP-3226, suggesting that the effects of NPY are mediated by NPY1R.

NPY, which is the most abundant neuropeptide in the mammalian brain mediates numerous physiological functions, including: orexigenic (increasing appetite) effect, neuroendocrine regulation, reducing anxiety, stress and pain perception, affecting the circadian rhythm and memory, lowering blood pressure, and controlling epileptic seizures^51–61^. The circulatory levels of NPY are reported to be elevated in hypertensive subjects, obesity, preeclampsia, and in some malignancies such as neuroblastoma and Ewing sarcoma due to high neuropeptide synthesis within tumor tissues^62–66^. NPY exerts most of its actions through at least five different GPCRs, NPYY1-5^67^. Among them, NPY1R is the only receptor with a high incidence in human breast carcinomas, however it is unclear whether the NPY is locally synthesized in breast tissue or endogenously expressed by breast cancer cells. NPY1R has been reported to be expressed in 85% of the primary human BC compared to normal breast tissue which only expresses the NPY2R receptor subtype^68^. Because of the high NPY1R expression in BC, it has been explored as a promising probe in cancer diagnosis^68^. Radiotracers for NPY1R-targeted BC imaging have been synthesized and used in several studies^68–71^. However, our studies find that NPY1R expression is high only in LumA BC but not in other subtypes. Furthermore, its expression is downregulated in the endocrine-resistance setting. Therefore, our results suggest that NPY1R may not be a good probe for cancer diagnostics, but rather to predict endocrine sensitivity and treatment response.

The significance of hyperphosphorylated status of NPY1R in LumA BC and its hypophosphorylation in other BC subtypes is not clear at this point but needs further evaluation. For class A GPCRs like NPY1R, agonist binding exerts the downstream signaling via the receptor coupling to G proteins^72^. This transient signaling is followed by desensitization of the GPCR by phosphorylation by GPCR kinases (commonly known as GRKs) followed by endocytosis^73^. In most cases, GPCRs are dephosphorylated and recycled back to the plasma membrane ready to bind to available agonist^74^. In this sense, hyperphosphorylation of NPY1R in LumA may indicate the presence of functional receptor. Because NPY1R mediates action of NPY on reducing E2-stimulated growth in ER+ BC cells, presence of functional receptor may indicate favorable outcome in patients. However, whether phosphorylation status of NPY1R predicts endocrine sensitivity needs to be assessed in future studies.

A functional interplay between estrogen and NPY1R has been shown in the ER+ BC cell line, where estrogen has been found to increase NPY1R expression, which in turn negatively regulated E2-stimulated cell proliferation^75^. Our results showing the inhibitory effects of NPY on E2-stimuated cell growth in ER+ BC cells is also consistent with a previous report^75^. These results also explain why higher expression of NPY1R is a favorable predictor of outcomes in ER+ BC. A higher expression of NPY1R should allow circulating NPY to inhibit E2-stimulated cell growth in tumors. However, it is not known how these effects can be leveraged to develop new therapeutics to improve efficacy of endocrine therapy in patients.

In ER+ BC, endocrine therapy is the most effective treatment option, but its effectiveness is limited by high rates of de novo resistance and resistance acquired during treatment^76^. Our studies highlighted the previously unknown role of NPY1R as a biomarker in the endocrine resistance setting where the receptor is downregulated. NPY1R gene expression was also found to be downregulated in patients resistant to aromatase inhibitors compared to responders^77^. These clinical data confirm our findings in preclinical models of endocrine resistance. More recently, cyclin D/cyclin-dependent kinases 4 and 6 inhibitors (CDK4/6i) have shown to be effective in overcoming endocrine resistance and are used in combination with endocrine therapy to increase the survival advantage of metastatic ER+ BC patients^78^. Despite having better clinical outcomes, acquired resistance to CDK4/6i presents a major clinical challenge^78–80^. The role of NPY1R as a biomarker of resistance to the combination of endocrine therapy and CDK4/6 inhibitors is unknown and should be investigated in the future.

## Conclusions

In summary, we have found NPY1R to be highly expressed and hyperphosphorylated GPCR in LumA breast tumors of patients. Our results demonstrate NPY1R expression as a predictor of endocrine sensitivity ER+ BC and long-term outcomes in patients. Future studies targeting NPY1R will further elucidate the role of NPY1R as a novel drug target in ER+ BC.

## Supplementary Information

Supplementary Legends.

Supplementary Figure 1.

Supplementary Figure 2.

Supplementary Figure 3.

Supplementary Figure 4.

Supplementary Figure 5.

Supplementary Figure 6.

Supplementary Figure 7.
