# Supplementary material for: NPY1R exerts inhibitory action on estradiol-stimulated growth and predicts endocrine sensitivity and better survival in ER-positive breast cancer
Source: Sci Rep. 2022 Feb 4;12:1972. doi: 10.1038/s41598-022-05949-7 (PMC8817007; doi:10.1038/s41598-022-05949-7)
Supplement: Supplementary file 1 — Supplementary Legends. [file 41598_2022_5949_MOESM1_ESM.docx]

**Supplementary Figure 1. NPY1R expression across TCGA cancer cohort obtained from human protein atlas (HPA).** NPY1R RNA-seq expression reported as median FPKM (number Fragments Per Kilobase of exon per Million reads) in 17 cancer types generated by The Cancer Genome Atlas (TCGA). Normal distribution across the dataset is visualized with box plots, shown as median and 25th and 75th percentiles. The outlier (above or below 1.5 times the interquartile range) shown as points.

**Supplementary Figure 2. BC subtype specific expression of NPY1R obtained from METABRIC study.** NPY1R RNA expression in METABRIC BC patient dataset (n=1,758) plotted across different molecular subtypes, LumA (n=679), LumB (n=461), HER2 (n=220), Basal (n=199), and Claudin-low (n=199). * indicates p < 0.05, One-way ANOVA, Tukey test.

**Supplementary Figure 3. NPY1R gene expression across BC cell lines obtained from the Cancer Cell Line Encyclopedia (CCLE).** RNA-seq data (log2, RPKM) for 50 BC cell lines were extracted from the Broad Institute CCLE database. The cell lines were grouped based on PAM50 subtype and the NPY1R mRNA expression was plotted as a double gradient heat map using GraphPad Prism version 7.04.

**Supplementary Figure 4. Endocrine therapy downregulates NPY1R gene expression in ER+ BC cells.** MCF7 and T47D cells grown in charcoal-stripped media were treated with Estradiol (E2), estrogen deprivation (ED), or ED + tamoxifen (Tam). RNA was extracted for RNA-Seq analysis, and the data was plotted as Reads Per Kilobase of transcript per Million mapped reads (RPKM) from one experiment.

**Supplementary Figure 5. NPY1R protein expression is impaired in endocrine-resistant derivatives of ER+ BC cells *in vitro*.** The densitometric quantitation of the relative intensity of NPY1R to parental cells (from three independent experiments) and representative full-length blots are presented. For the densitometric analysis, lack of a band for NPY1R in EDR and TamR of MCF7 and T47D yielded density of 0, which could not be evaluated for statistically significant difference compared to Parental cells. * indicates p < 0.05 by One-way ANOVA, Dunnett’s test (n=3-4).

**Supplementary Figure 6. BIBP-3226 has no significant effects on non-E2-stimulated cell growth in MCF7 and T47D cells.** MCF7 and T47D cells were treated with either vehicle (0.1% DMSO) or 1µM BIBP-3226 and then plated in 96-well plates. The cell proliferation was determined using MTT assay after 72 hours.

**Supplementary Figure 7. BC specific survival (BCSS) of METABRIC dataset of BC patients with high and low expression of NPY1R.** Subtype specific survival analyses of NPY1R regarding BCSS using METABRIC dataset. Red: high expression group; blue: low expression group. p-value was log-ranked. Auto-selected best cutoff was used.
