# Supplementary figures and images for: NPY1R exerts inhibitory action on estradiol-stimulated growth and predicts endocrine sensitivity and better survival in ER-positive breast cancer
Source: Sci Rep. 2022 Feb 4;12:1972. doi: 10.1038/s41598-022-05949-7 (PMC8817007; doi:10.1038/s41598-022-05949-7)

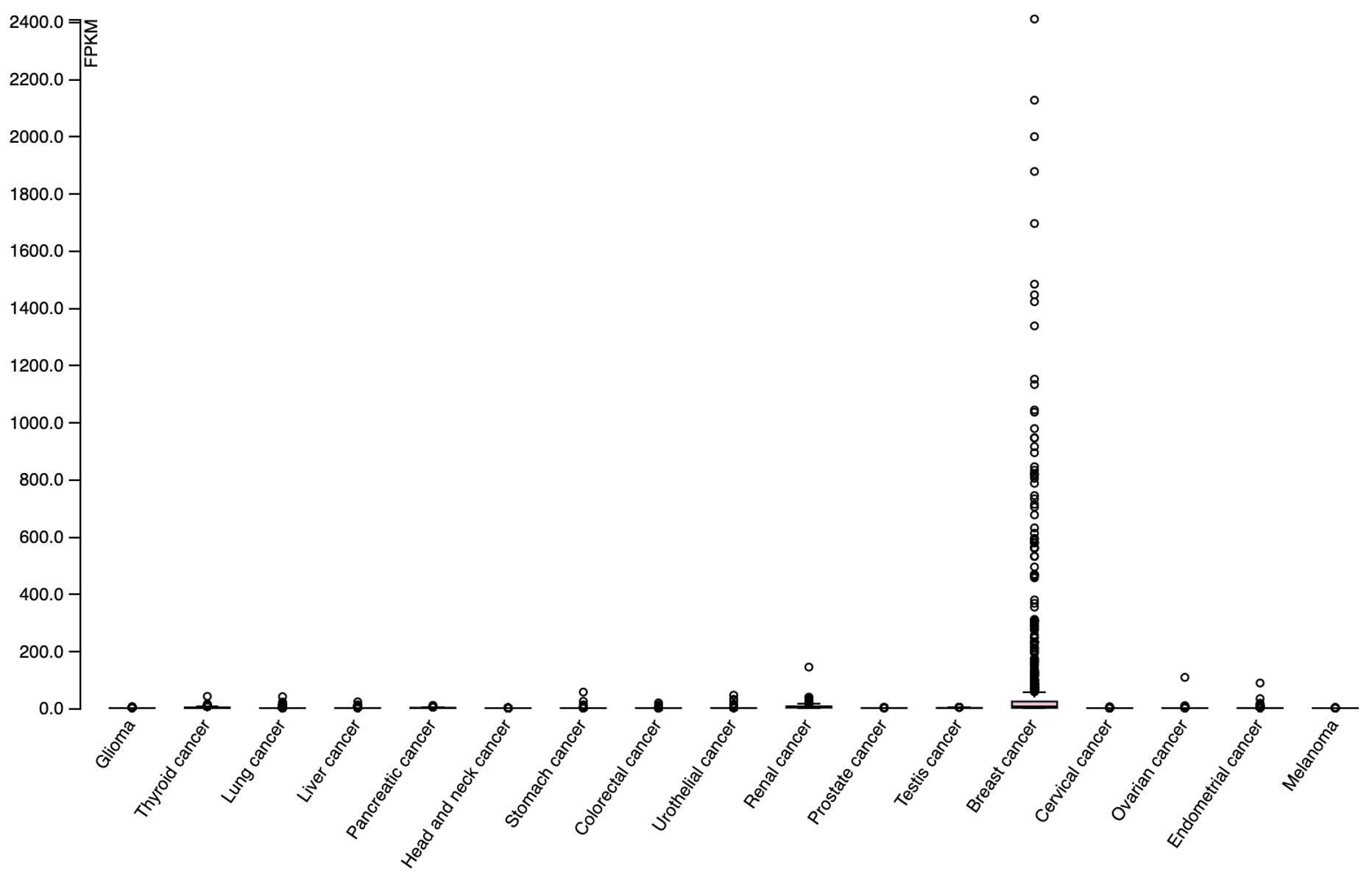

Supplement: Supplementary file 2 — Supplementary Figure 1. [file 41598_2022_5949_MOESM2_ESM.jpg]

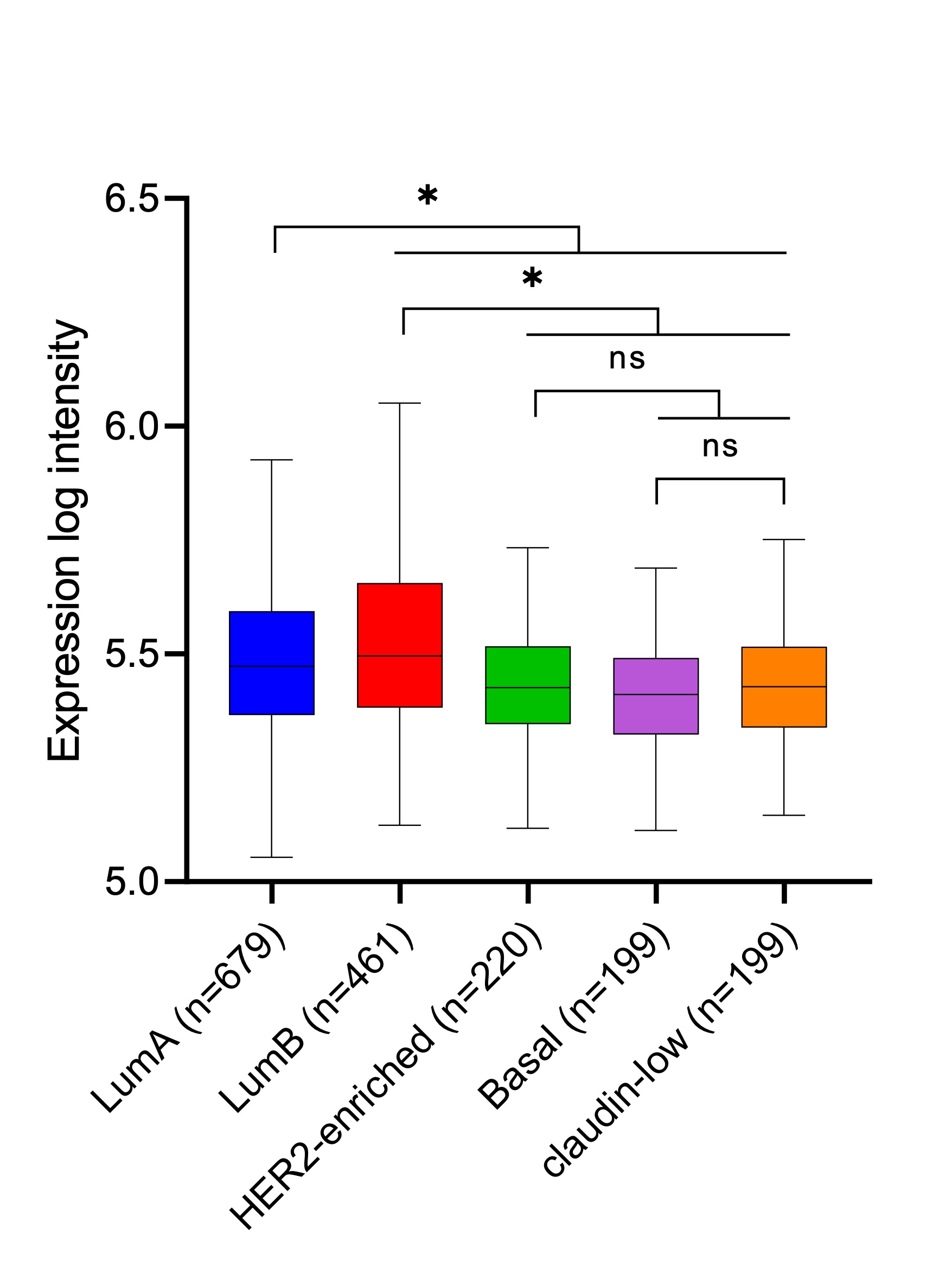

Supplement: Supplementary file 3 — Supplementary Figure 2. [file 41598_2022_5949_MOESM3_ESM.jpg]

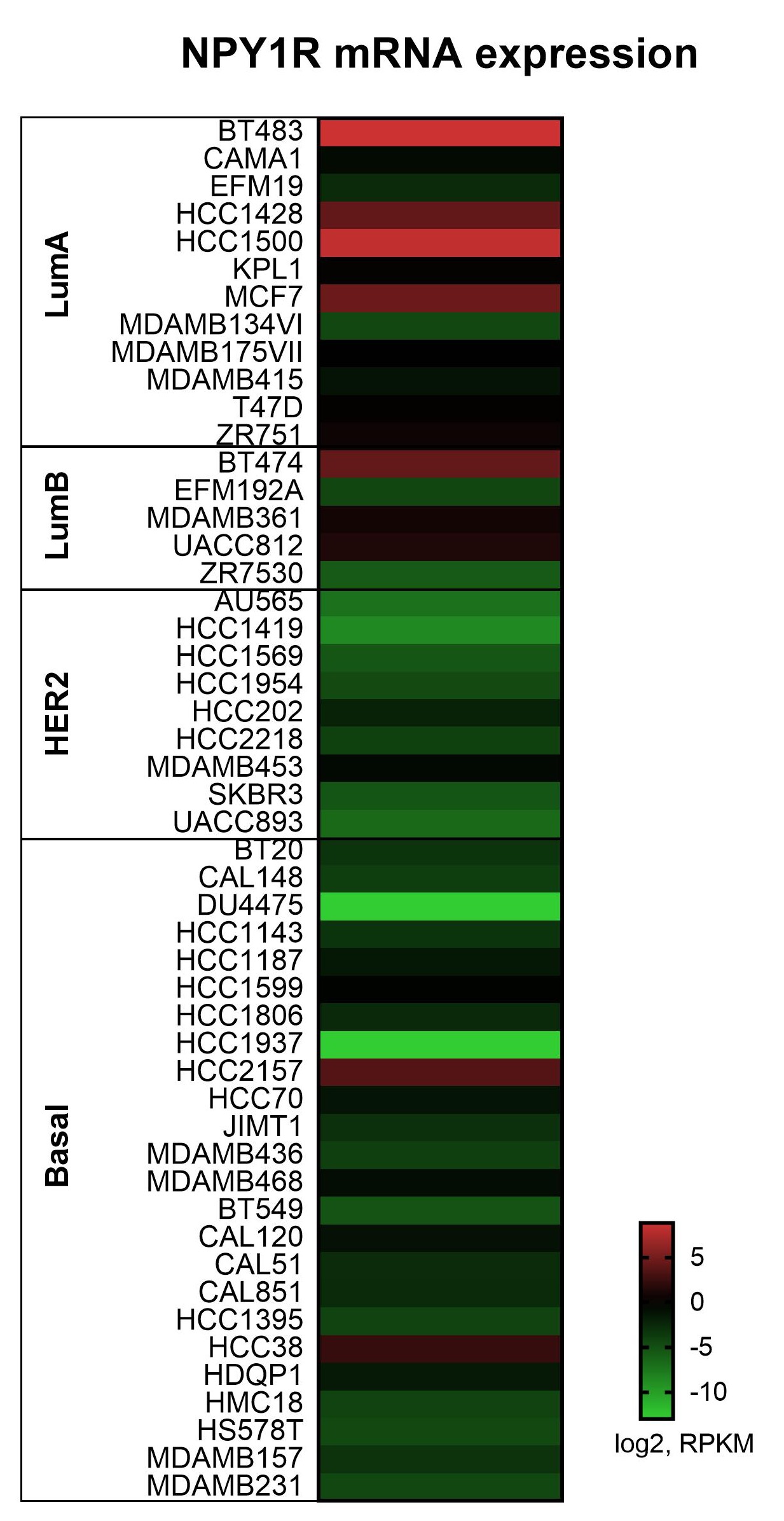

Supplement: Supplementary file 4 — Supplementary Figure 3. [file 41598_2022_5949_MOESM4_ESM.jpg]

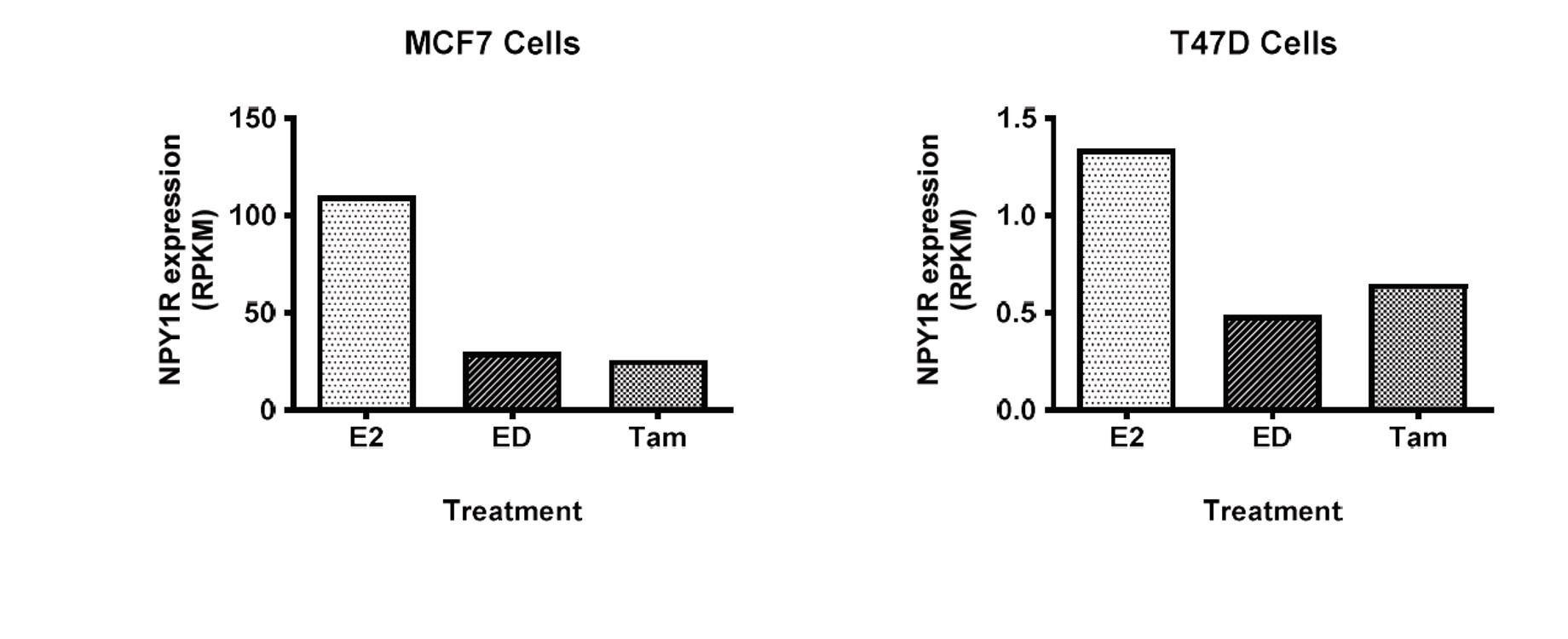

Supplement: Supplementary file 5 — Supplementary Figure 4. [file 41598_2022_5949_MOESM5_ESM.jpg]

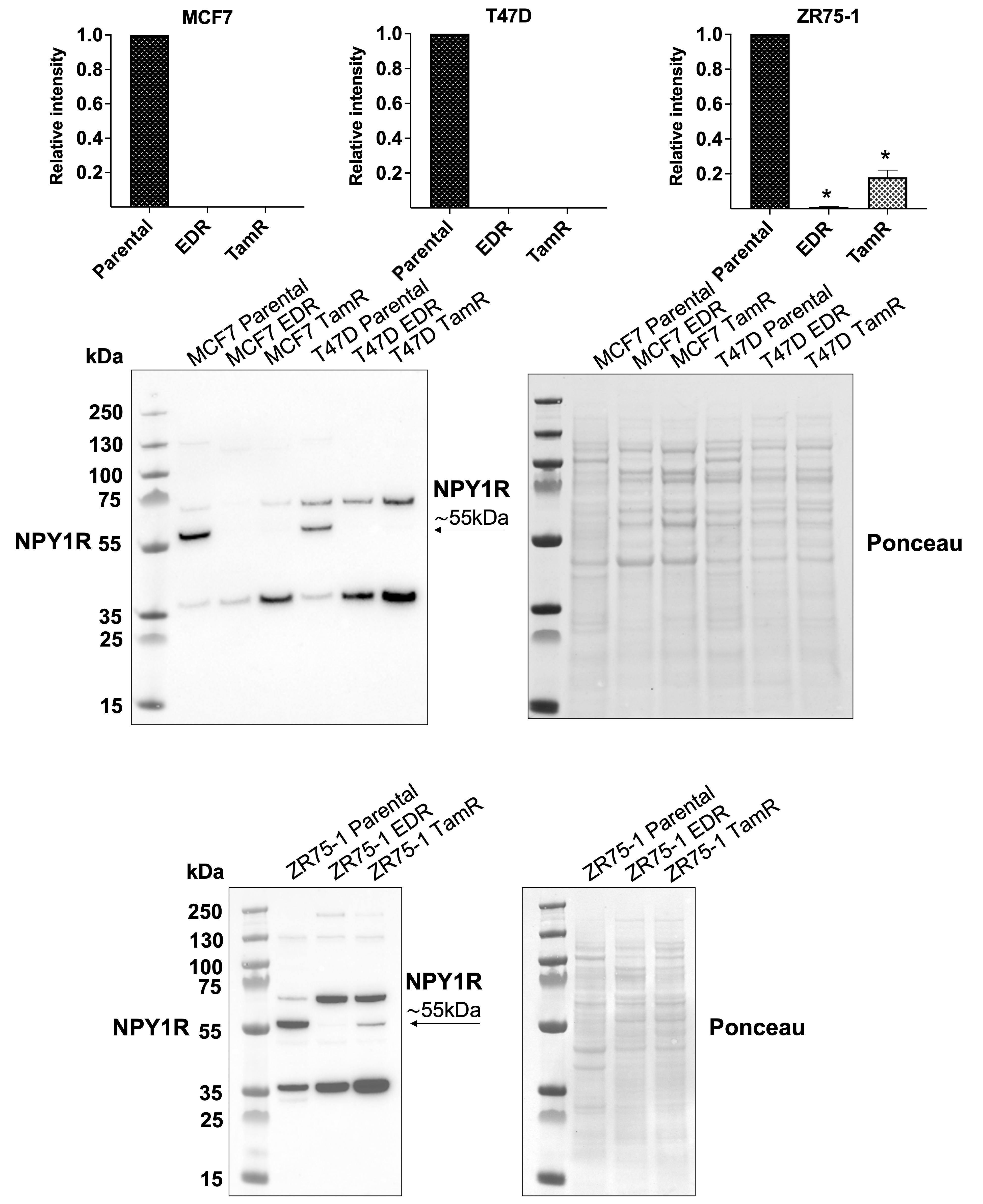

Supplement: Supplementary file 6 — Supplementary Figure 5. [file 41598_2022_5949_MOESM6_ESM.jpg]

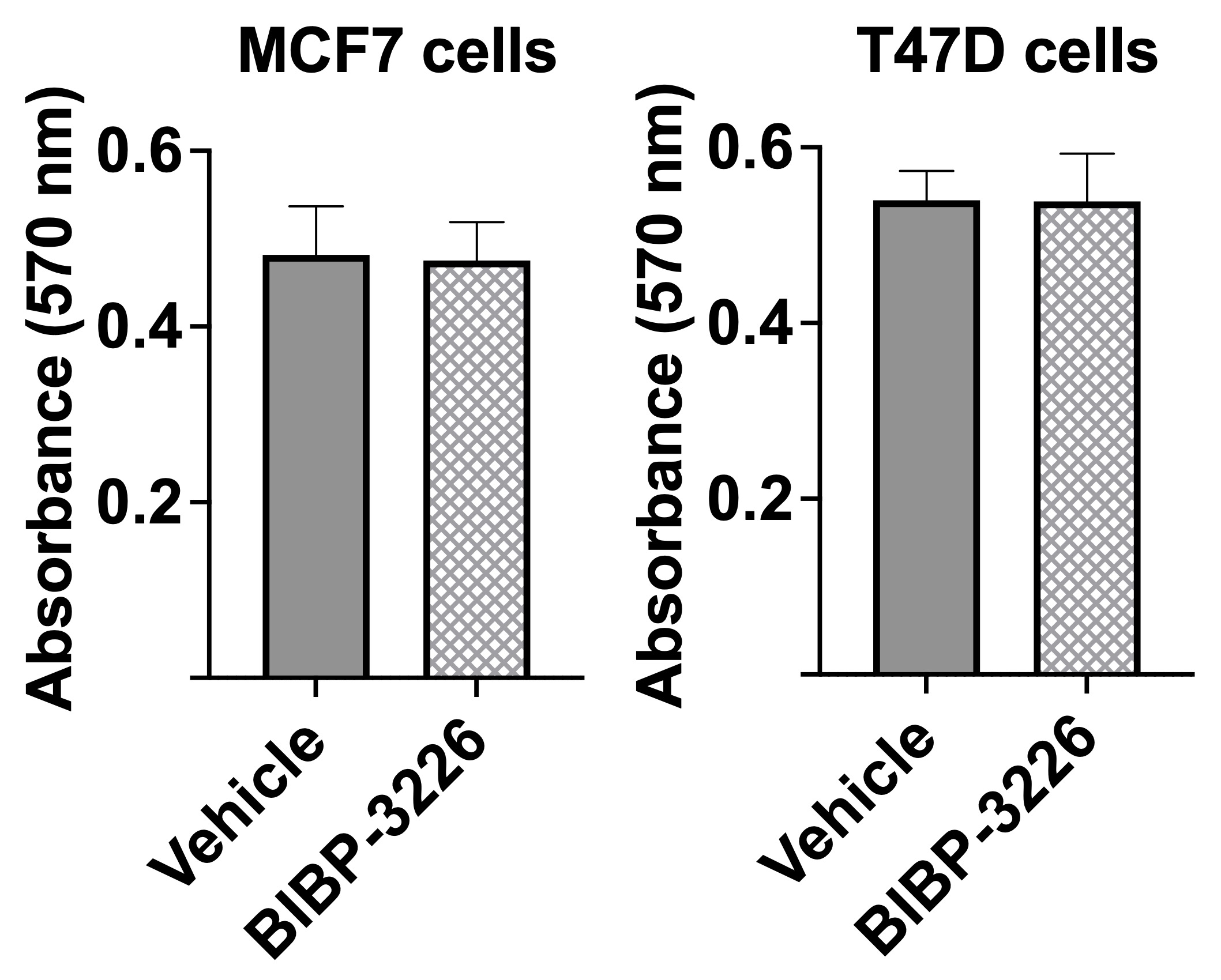

Supplement: Supplementary file 7 — Supplementary Figure 6. [file 41598_2022_5949_MOESM7_ESM.jpg]

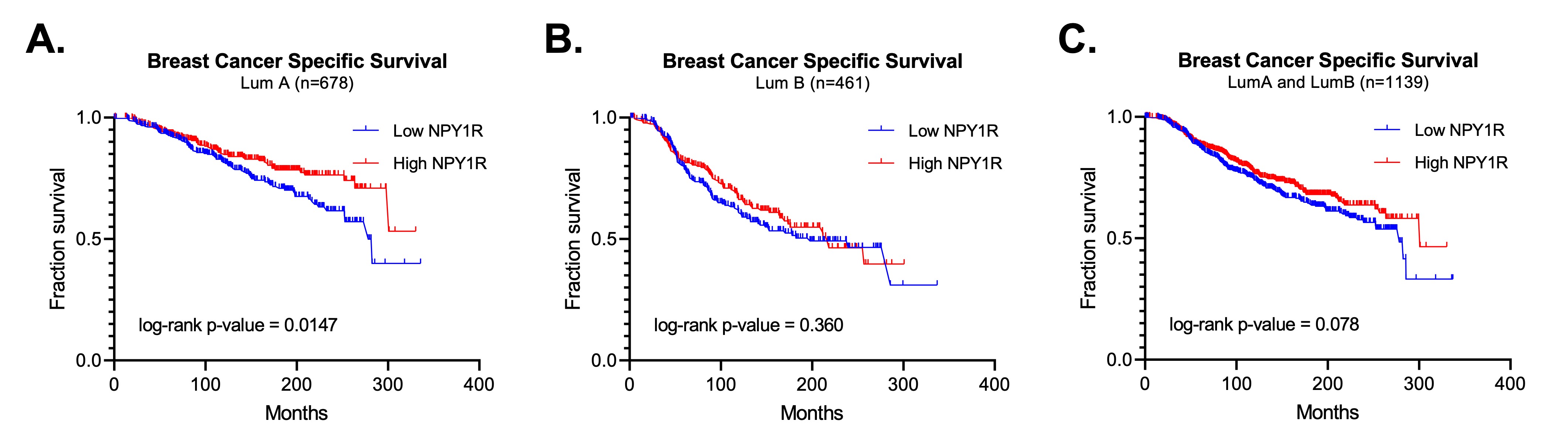

Supplement: Supplementary file 8 — Supplementary Figure 7. [file 41598_2022_5949_MOESM8_ESM.jpg]
